# Recent insights into the biological functions of hesperidin

**DOI:** 10.17179/excli2025-8730

**Published:** 2025-08-18

**Authors:** Chanung Park, Ji Hyun Yoo, Sang Un Park

**Affiliations:** 1 Department of Crop Science, Chungnam National University, 99 Daehak-ro, Yuseong-gu, Daejeon, 34134, Korea; 2 Department of Oriental Medicine and Pharmaceutical Science, Joongbu University, 201 Daehak-ro, Chubu-myeon, Geumsan-gun, Chungcheongnam-do 32713, Korea; 3 Department of Smart Agricultural Systems, Graduate School, Chungnam National University, Daejeon 34134, Korea

## ⁯⁯⁯⁯⁯⁯

Hesperidin (3,5,7-trihydroxyflavanone 7-rhamnoglucoside) is a β-7-rutinoside of hesperetin (4′-methoxy-3′,5,7-trihydroxyflavanone) (Sharma et al., 2021[57]). It is a flavonoid and, thus, one of the much extended flavanones, which are among the most abundantly existing phenol compounds in plants (Hajialyani et al., 2019[16]; Rahmani et al., 2023[52]). Hesperidin and its aglycone derivatives are major compounds in citrus fruits of the Rutaceae family: orange (*Citrus sinensis*), grapefruit (*Citrus paradise*), tangerine (*Citrus reticulata*), lime (*Citrus aurantifolia*) and lemon (*Citrus limon*) (Lee and Han, 2013[37]; Phucharoenrak et al., 2022[50]; Rodrigues and Pintado, 2024[54]; Wu et al., 2007[62]; Xu et al., 2024[63]). Their abundance in citrus fruit depends on the plant variety, fruit tissue type, climate and the stage of fruit ripening (Gattuso et al., 2007[12]; Pyrzynska, 2022[51]).

Hesperidin possesses biological and pharmacological activities, such as antioxidant, anti-inflammatory, antibacterial, antiviral, anti-allergic, anticancer, cardiovascular protective, and neuroprotective properties (El‐Shiekh et al., 2025[11]; Kowalczyk, 2024[33]; Ma et al., 2024[42]).

Both pre-clinical and clinical studies have demonstrated the beneficial effects of hesperidin on various diseases, such as bone, cardiovascular, neurological, respiratory, digestive and urinary tract diseases (Hosawi, 2023[20]; Ji et al., 2024[27]). Previously, the chemical composition and major skin application areas for hesperidin were described as follows: (i) anti-ageing and skin barrier protection, (ii) UV radiation damage, (iii) hyperpigmentation and depigmentation conditions, (iv) wound healing, and (v) skin cancer and other cutaneous diseases (Rodrigues and Pintado, 2024[54]). The novelty of this work is based on the exploitation of the encouraging skincare and elasticity applications of hesperidin, which represents a sustainable ingredient in the circular economy. Here, we discuss important recent studies that have been conducted to assess the health-promoting and pharmacological properties of hesperidin (Table 1(Tab. 1); References in Table 1: Abu-Khudir et al., 2023[1]; Ahmed et al., 2025[2]; Arneth et al., 2025[3]; Artanti et al., 2024[4]; Ashry et al., 2023[5]; Cao et al., 2023[6]; Choudhury et al., 2025[7]; Çınar et al., 2025[8]; Deng et al., 2024[9]; Durgun et al., 2023[10]; Geervani et al., 2025[13]; Gou et al., 2024[14]; Guo et al., 2024[15]; Han et al., 2023[17]; Hashimoto et al., 2025[18]; He and Liao, 2025[19]; Hu et al., 2023[21]; Huang et al., 2024[22][23]; Jamal et al., 2024[24]; Jamali et al., 2024[25]; Jayaraman et al., 2024[26]; Kaewngam et al., 2024[28]; Kale et al., 2024[29]; Karacaer et al., 2023[30]; Khezri et al., 2024[31]; Kim et al., 2023[32]; Kumar et al., 2024[34]; Kuşi et al., 2025[35]; Laila et al., 2023[36]; Li et al., 2024[38], 2025[39]; Liu et al., 2024[40]; Lou et al., 2024[41]; Maquera-Huacho et al., 2023[43]; Megahed et al., 2024[44]; Mohamed et al., 2024[45]; Nasehi et al., 2023[46]; Oh et al., 2023[47]; Ozyigit et al., 2024[48]; Park and Yu, 2024[49]; Reddy et al., 2024[53]; Shaban et al., 2025[55]; Shabani et al., 2024[56]; Shirodkar et al., 2024[58]; Talebi et al., 2024[59]; Tang et al., 2025[60]; Wang et al., 2025[61]; Zarein et al., 2023[64]; Zhang et al., 2024[65], 2025[66]).

## Notes

Chanung Park and Ji Hyun Yoo contributed equally as first author.

## Declaration

### Acknowledgments

This research was supported by the Bio & Medical Technology Development Program of the National Research Foundation (NRF) funded by the Ministry of Science, ICT & Future Planning (2016M3A9A5919548).

### Conflict of interest

The authors declare no conflict of interest.

## Figures and Tables

**Table 1:**
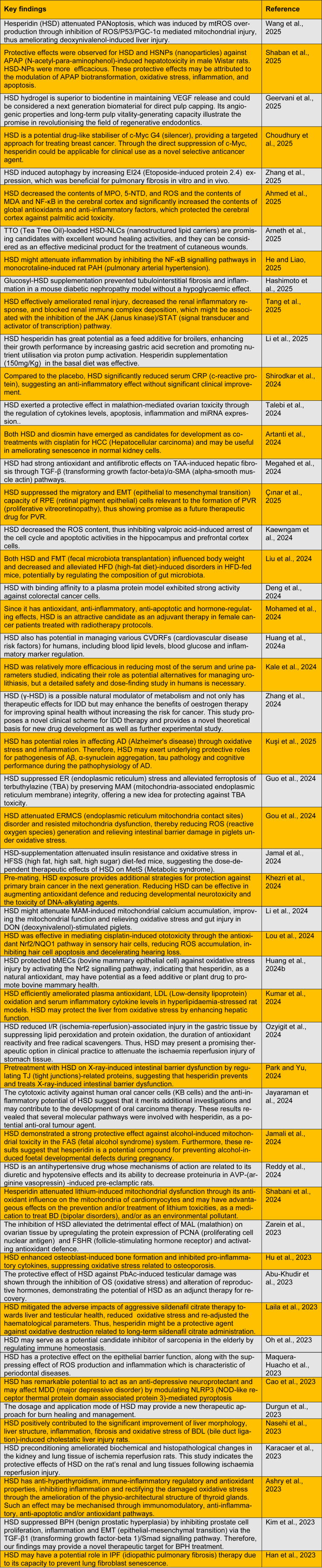
Recent studies on the biological and pharmacological activities of hesperidin
